# A 10-state investigation of AD/ADRD-capable assisted living regulations using a comprehensive dementia care model

**DOI:** 10.3389/frdem.2026.1789887

**Published:** 2026-07-16

**Authors:** Margaret Manchester, Alice Boughrum, Victoria Helmly, Olusheun Olupitan, Jalayne Arias

**Affiliations:** 1 Department of Health Policy and Management, School of Public Health, Georgia State University, Atlanta, GA, United States; 2 Georgia Health Policy Center, Georgia State University, Atlanta, GA, United States; 3 School of Law, Georgia State University, Atlanta, GA, United States

**Keywords:** Alzheimer's disease care, assisted living, cognitive impairment, dementia care, long-term care, memory care, assisted living regulation, memory care model

## Abstract

**Introduction:**

State regulation is central in shaping dementia care practices in assisted living facilities. Yet, there is limited systematic evidence examining how regulatory requirements vary and affect residents across states. Additionally, regulatory frameworks governing the training requirements and staff presence of ALFs play a vital role in shaping ALFs' dementia care capacity.

**Methods:**

This article reports the results of a legal mapping study that evaluated the variation of assisted living facility (ALF) memory care regulations across 10 states. We compared the regulations of these 10 selected states to the "gold standard" Dementia Care Practice Recommendations, a comprehensive memory care model compiled by the Alzheimer's Association (Fazio et al., 2018).

**Results:**

Of the care model's nine recommendations, regulations aligned most to the following: 1. Supportive and Therapeutic Environment (84% average alignment across 10 states), 2. Information Education and Support (74% average), and 3. Ongoing Care (70% average). States with regulation in highest alignment to the care model included California, Missouri, and Minnesota. Regulations were least aligned with the following recommendations: 7. Person-Centered Care (55% average), 8. Detection and Diagnosis (51% average), and 9. Medical Management (50%). States with regulation in lowest alignment to the care model included Texas, Georgia, and Connecticut.

**Discussion:**

Overall, inconsistencies in state alignment to the Care Model also underscore the absence of a standardized enforcement mechanism across assisted living systems. Additional evidence is needed to link regulatory standards to actual measured changes in state-level care coordination practice in the ALF setting. Target areas for regulation change may also exist outside of mechanisms that govern the staff and services directly provided by ALFs. Methods of this study are building blocks from which researchers may systematically collect and analyze regulations data for future across-state LTSS studies.

## 1 Introduction

Long-term care residential facilities experience high rates of cognitive impairment and dementia among their residents. In 2020, 42% of residential care facilities, including assisted living (ALFs), reported their residents had Alzheimer's Disease, or, a related dementia diagnosis (“Biennial Overview of Post-Acute and Long-Term Care in the United States: Data from the National Post-Acute and Long-Term Care Study | Data | Centers for Disease Control Prevention, 2025”). The National Survey of Residential Care Facilities (NSRCF) also reported that 70% of ALF residents have some form of cognitive impairment (Zimmerman et al., 2003, 2014). High rates of cognitive impairment among residents highlight the need for basic memory care training for all care workers in ALFs. Residents living with dementia and other forms of cognitive impairment can also require additional and sometimes more complicated care, but may not have placement within an ALF's memory care unit or full facility.

In the absence of adequate memory care training and staffing standards, residents with cognitive impairment may be at heightened risk for unmet care needs, avoidable adverse events, and diminished quality of life. Regulatory frameworks governing training requirements and staff presence therefore play a central role in shaping dementia care capacity within ALF settings. However, federal regulation of long term services and supports does not include ALFs. Therefore, different state regulations establish standards that may affect the quality of care that residents living with cognitive impairment or dementia experience in these settings. Despite the centrality of state regulation in shaping dementia care practices in ALFs, there is limited systematic evidence examining how relevant regulatory requirements vary across states. Given evidence of an aging population (United States Census, 2025), and aforementioned lack of federal regulations that affect training and direct care staff presence in ALFs, it is imperative to evaluate the state-level variations in these regulations.

ALFs are a residential long-term care option for older adults not requiring the level of medical care provided in federally regulated nursing homes. Researchers and regulators also refer to ALFs as residential care facilities, community care homes, or assisted living communities, though service descriptions remain consistent. These differ from non-residential long-term services, such as adult day centers or home and community-based services, which do not provide 24-h residential care. Generally, the ALF categorization includes residential programs that have not been federally licensed as a nursing home but provide care in activities of daily living (ADLs) and can respond to unscheduled needs of residents (Zimmerman and Sloane, 2007). ALFs are a model of facility-based living that provides diversity in choices of service, resident lifestyle, and independence. Basic health care assistance and services are provided as needed, often by aides (uncertified), CNAs, or LPNs. Sometimes, these direct care workers are overseen by supervisory, higher-accredited personnel such as an NP or RN (Chidambaram et al., 2024).

As the aging population increases, there is increasing complexity in residents' needs. Some ALFs have absorbed this responsibility and are delivering services that extend beyond traditional assisted living models. Sometimes, they may even resemble nursing home–level care without the federal certification (Zimmerman et al., 2024, 2003). However, unlike nursing homes, state governments and agencies regulate ALFs so there is considerable state-by-state variation in the guaranteed services and staffing requirements. Additionally, the integration and descriptions of memory care in ALF regulation largely vary by state. In 2024, Smith et al. found that across 50 states and D.C., 350 different licenses and certifications exist (Smith et al., 2023). While some states require additional certification or licensing for an ALF to provide memory care, others are required only to disclose and describe whether memory care is provided (Carder, 2017).

This variation between state ALF mandates may have consequences for the quality of services provided and residents' health outcomes. Specifically, states often use responsive regulation (Ayres and Braithwaite, 1992) for regulation of a variety of long term care services and supports. Following this method, best practices are first encouraged with guidance before evidence that necessitates implementation of formal rules for compliance is produced. This explains existing variance in the presence and specificity of ALF regulations across states but also justifies integration of evidence-based care practices into ALF regulation at the state level.

This article reports the results of a legal mapping study that evaluated the variation of assisted living facility memory care regulations across 10 states to a “gold standard” comprehensive memory care model compiled by the Alzheimer's Association (Fazio et al., 2018b). For brevity, this is referenced as the Care Model throughout. The purpose of this study was to investigate where ALF regulation varies, to what extent regulations are consistent with best care practices.

## 2 Materials and methods

The team used legal epidemiology to conduct a systematic content analysis examining regulatory standards of 10 select states. This study included a systematic collection and review of both state statutes and administrative codes that establish memory care services and direct care staff training requirements for ALFs. Regulation collection was limited to licensed or certified assisted living facilities, communities or facilities with more than four beds, primarily servicing older adults, and those not exclusively licensed as nursing homes (Table 1). Regulations for states with separate licensing or certification for memory care units within ALFs were included in analysis.

**Table 1:** Facility types and specified regulations included in analysis, by state.

State	Facility type included in analysis	Abbreviated cite	Portions specific to memory/alzheimer's care within ALFs	Historical versions used in analysis
California	Residential care facilities for the elderly	Cal. Code Regs. Tit. 22, §§ 87100-87468	Article 12. Dementia, §§87707-87731.4	Eff. 3-5-2008 to 8-8-2022
		Cal. Health & Safety Code §§ 1569.2–1569.69	§§ 1569.4(I), § 1569.698(a)(1-3), (b)(2-5), (d)	Eff. 1-1-2017 to current
Connectiut	Assisted living services agencies	19-13-D105 Conn. Agencies Regs. § 19-13-D105	-	Eff. 6-29-2001 to current
		Conn. Gen. Stat. §§ 19a-562-19a562b, 19a563, 19a-564	§ 19a-562 Dementia special care units or programs. § 19a563 Nursing homes^**^ and dementia special care units. Infection prevention and control specialist. Definitions. Requirements. §19a-564. Assisted living services agencies. Licensure. Dementia special care approval.	Eff. 10-1-2007 to 7-5-2021
Georgia	Assisted living communities, personal care homes	Ga. Comp. R & Regs. § 111-8-63.01-111-8-63.34	§§ 63.09 Workforce Qualifications, Training and Staffing. §§ 63.18 Precautions for Residents at Risk of Elopement. §§ 63.19 Additional Requirements for Certified Memory Care Centers.	Eff. 1-2-2012 to 9-12-2021
		Ga Comp. R. & Regs. § 111-8-62.01-111-8-62.34	§§ 111-8-62.19 Additional Requirements for Certified Memory Care Centers	Eff. 1-8-2023 to 9-12-2021
Indiana	Residential care facility	410 In. Admin. Code 16.2-5	§5-1.4 Personnel.	Eff. 3-1-2003 to 11-14-2025 (readoption filed)
Kentucky	Assisted living communities, contiuing care retirement communities, personal care homes^*^	910 Ky. Admin. Regs. 1:240	–	Eff. 5-3-2019 to 2-12-2025
		902 Ky. Admin. Regs. 20:036	–	Eff. 7-29-2020 to 7-29-2024
		Ky. Rev. Stat. §§ 194A.700-194A.719	§§ 194A.719 In-service education for staff and management	Eff. 7-29-2017 to 8-13-2022
		Ky. Rev. Stat. §§ 216.510-216.765, 216B.015,216B.072, 216B.160	§§ 216.595 Requirements for assisted-living communities and long-term care facilities claiming to provide special care for persons with Alzheimer's disease or other brain disorders; waiver on building requirements to address specific needs. §§ 216.B.072 Training for staff of long-term care facilities treating persons with Alzheimer's disease or related disorders.	Eff. 8-14-2018 to 8-13-2022
Minnesota	Assisted living facilites, boarding care homes	Minn. Stat. § 144G^***^	§144G.63 Orientation and Annual Training Requirements.	Eff. 1-1-2007 to 8-1-2021
		Minn. R. 4655^***^	–	Eff. 1-19-2005 to current^**^
Missouri	Assisted living facilities	19 Mo. Code of State Regs. 30-82	§§ 30-82.010(1)(B) General Licensure Requirements	Eff. 4-22-2020 to 11-28-2021
		19 Mo. Code of State Regs. 30-86	§§30-86.047(63-64) Administrative, Personnel, and Resident Care Requirements for Assisted Living Facilities. §§ 30-86.042(20)(A-C) Administrative, Personnel and Resident Care Requirements for New and Existing Residential Care Facilities. §§30-86.045. Standards and Requirements for Assisted Living Facilities Which Provide Services to Residents with a Physical, Cognitive, or Other Impairment that Prevents the Individual from Safely Evacuating the Facility with Minimal Assistance.	Eff. 4-30-2007 to 11-30-2025
New Mexico	Assisted living facilities	N.M. Admin Code. § 7.8.2-7.8.2.70	§ 7.8.2.69 Memory Care Units.	Eff. 1-15-2010 to 4-16-2024
Texas	Assisted living facilities	26 Tex. Admin. Code § 553	§ 553.253(2),(3)(F) Employee Qualifications & Training. § 553.254. Training Requirements for Staff Providing Personal Care Services to a Resident With Alzheimer's Disease or a Related Disorder in a Facility that is Not an Alzheimer's Certified Facility. § 553.255. All Staff Policy for Residents with Alzheimer's Disease or a Related Disorder. § 553.301 Subchapter F. Additional Licensing Standards for Certified Alzheimer's Assisted Living Facilities.	Eff. 4-12-2019 to 8-31-2021
Virginia	Assisted living facilities	22 Va. Admin. Code ch 73	Part X. Additional Requirements for Facilities that Care for Adults with Serious Cognitive Impairments	Eff. 12-11-2017 to 8-14-2024

^*^Excludes parts of regulation specific to Specialized Personal Care Homes (SPCHs). i.e., those serving Intellectually Disabled or Developmentally Disabled.

^**^Nursing home subsections not included in analysis.

^***^Since August 1, 2021, Minnesota Dept. of Health has also adopted Chapter 144G and Regulation 4659, with expanded regulation specific only to assisted living facilities and assisted living facilities providing memory care.

The research team (MM, OO) first semi-randomly selected 10 states known to have ALF regulations active and effective in 2020. States were divided into five geographic regions (West, Midwest, South, Southwest, and Northeast). Selection was based on other branches of research interest within the broader team, with remaining states semi-randomly selected so that the final 10 included at least one state from each of the five regions. Final state selection included: California, Missouri, Minnesota, Indiana, Kentucky, New Mexico, Virginia, Texas, Georgia, and Connecticut.

Legal epidemiology is the “scientific study of law as a factor in the cause, distribution, and prevention of disease and injury of a population”(Wagenaar et al., 2023; Center for Public Health Law Research, 2025). This method, which includes legal mapping, supports a quantitative mapping of laws and qualitative analysis of the text within individual laws and regulations. Using legal mapping methods, we compared the 10 selected states' ALF regulations to the Alzheimer's Association's 2018 Comprehensive Dementia Care Practice Recommendations (Fazio et al., 2018b).

We methodically searched Westlaw and compiled state regulations mentioning either ALFs or memory care during January 2025 to May 2025. Retrieval of regulation was a semi-snowball process. Boolean search terms included (“assisted living” OR “residential care” OR “memory care” OR “dementia care” OR “Alzheimer's” “NOT nursing home”), then repeated adding (AND “training,” “AND “definitions,” “AND “direct care”). Regulations that included amendments effective after June 30, 2020 were excluded from the search. In these cases, historic versions that were active in 2020 were found using Westlaw or state websites. MM and OO also cross-referenced state websites to confirm accuracy of regulation, effective dates, and to identify the regulatory bodies responsible (state agency and/or department). The research team used legal mapping and a modified coding process to extract relevant information (or content) from the collected regulations. For extraction, the team used MonQcle, a web-based platform for the analysis of legal documents. Text on direct care staff training, required resident care standards for ALFs, required care standards for ALF memory care units, full descriptions and definitions of memory care, and a list of the capable services that ALF direct care workers were required to provide were all imported into MonQcle, then manually sorted. The sorting and coding process was guided by the Alzheimer's Association's 2018 Comprehensive Dementia Care Practice Recommendations (Fazio et al., 2018b). This Care Model contains nine recommendations, of which each contains five to 10 specified criteria per recommendation (Table 2). The Care Model contains 56 criteria total, of which each could be independently met by specific regulation content that described, matched verbatim, or otherwise identified the criterion. Presence of individual criteria within each recommendation were coded, and absence of criteria noted. Each individual criterion that was coded, regardless of frequency in a states' regulation, added 1 “point” to the states' overall score, with 56 maximum possible.

**Table 2:** Average State Alignment to Alzheimer's Association Comprehensive Care Recommendations; with Criteria.

Recommendation	Criteria	Average criteria alignment
Supportive and therapeutic environment (Calkins, 2018)	Create a sense of community within the care environment; Enhance comfort and dignity for everyone in the care community; Support courtesy, concern, and safety within the care community; Provide opportunities for choice for all persons in the care community; Offer opportunities for meaningful engagement to members of the care community	84%
Information, education, and support (Whitlatch and Orsulic-Jeras, 2018)	Provide education and support early in the disease to prepare for the future; Encourage care partners to work together and plan together; Build culturally sensitive programs that are easily adaptable to special populations; Ensure education, information, and support programs are accessible during times of transition; Use technology to reach more families in need of education, information, and support	74%
Ongoing Care (Prizer and Zimmerman, 2018; Scales et al., 2018)	Identify characteristics of the social and physical environment that exacerbate symptoms; Implement non-pharmacological person-centered practices; Recognize investment differences across settings; Adhere to administration protocols; Develop evaluation systems; Support dignity and choice in dressing and ADLs; Follow person-centered care practices; Address toileting, dining, and biological needs	70%
Staffing (Gilster et al., 2018)	Provide orientation and ongoing training; Develop systems for collecting information; Encourage communication and teamwork; Establish supportive leadership; Promote resident, staff, and family relationships; Evaluate systems for improvement	67%
Person-centered assessment and care planning (Molony et al., 2018)	Perform regular comprehensive assessments; Use assessments for education and relationship-building; Apply collaborative team approach; Use communication systems across providers; Encourage advance planning and awareness of care options	62%
Transitions and coordination of care (Hirschman and Hodgson, 2018)	Prepare and educate persons and caregivers on transitions; Ensure timely communication; Evaluate preferences and goals; Create strong interprofessional teams; Use evidence-based transition models	56%
Person-centered care (Fazio et al., 2018a)	Know the person living with dementia; Accept the person's reality; Support meaningful engagement; Build authentic relationships; Maintain supportive communities; Evaluate practices regularly	55%
Detection and Diagnosis (Maslow and Fortinsky, 2018)	Make brain health information available; Recognize cognitive impairment signs; Observe and track cognitive changes; Maintain detection procedures; Use brief mental status tests; Encourage diagnostic follow-through; Support understanding of diagnosis	51%
Medical Management (Austrom et al., 2018)	Use holistic person-centered care; Understand provider roles; Address comorbidities; Encourage non-pharmacologic interventions first; Collaborate on care plans for crises; Encourage early end-of-life discussions	50%

## 3 Results

**Figure 1:**
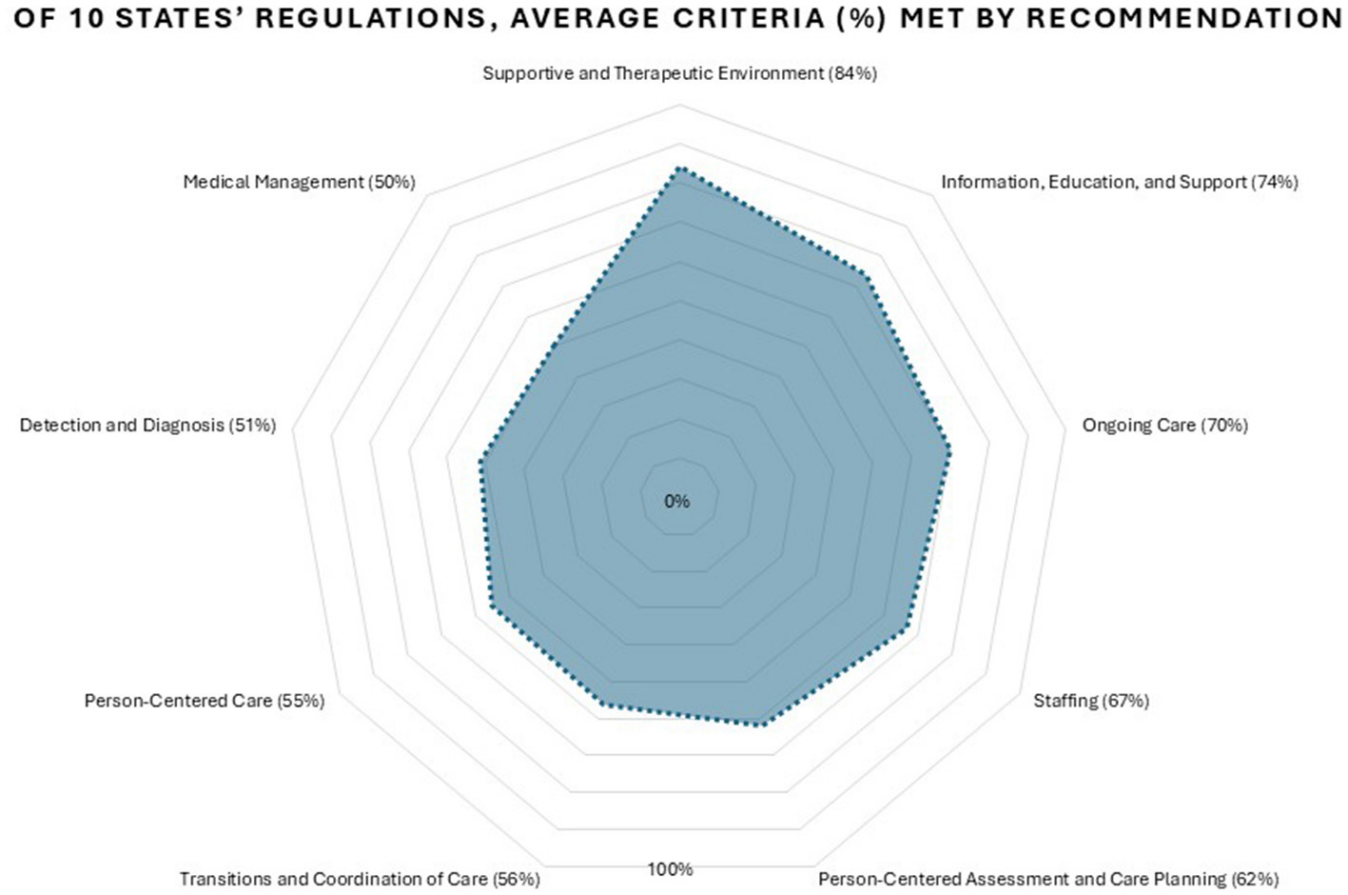
Of 10 states analyzed, average criteria alignment scores (%), by each recommendation in the comprehensive care model.

**Figure 2:**
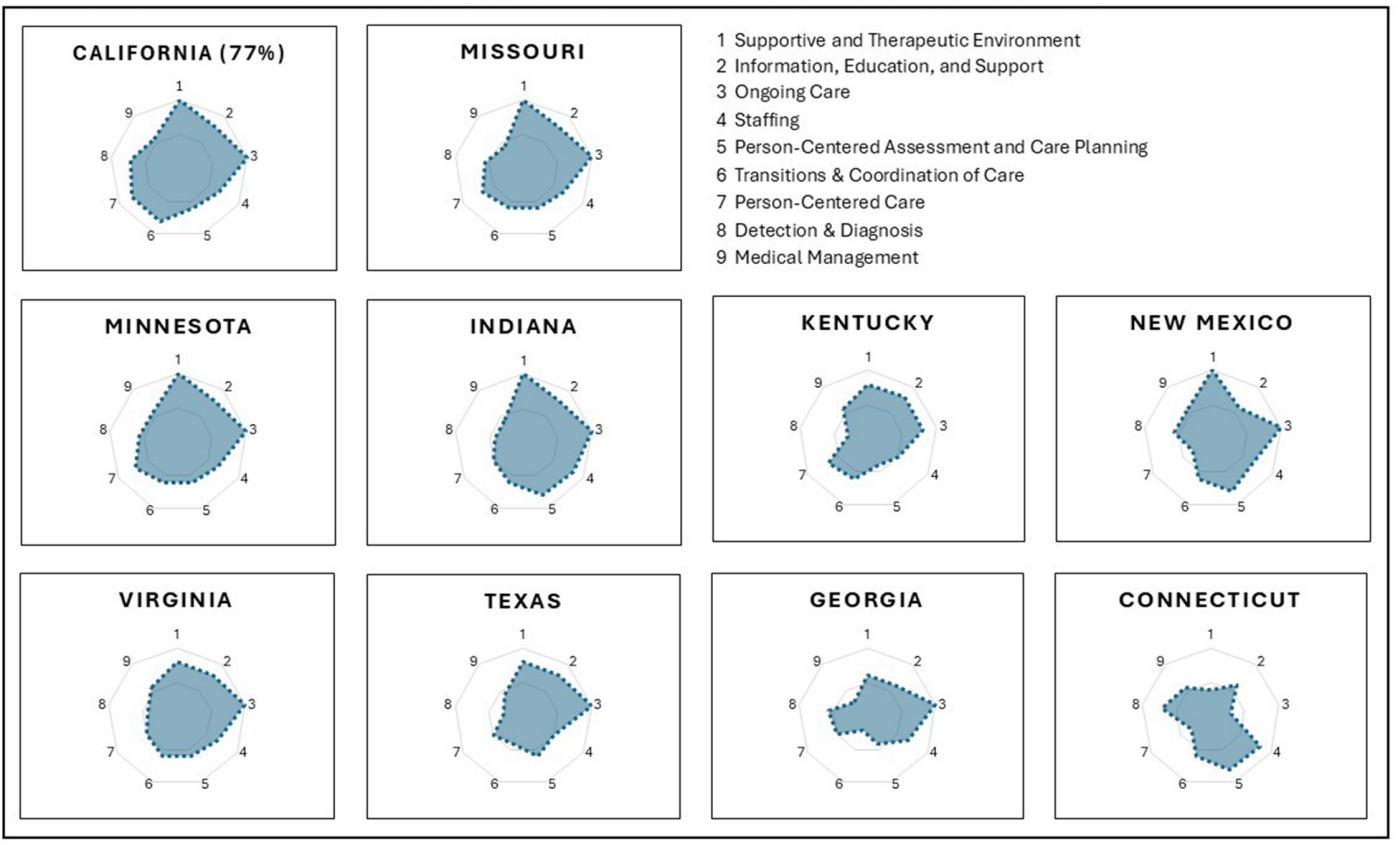
Alzheimer's association comprehensive care criteria met, by state total alignment and recommendation.

A list of the individual state regulations included in analysis can be viewed in Table 2. Both statues and administrative code were included in the analysis. First, we report on individual Care Model recommendations with the highest alignment (Figure 1). These recommendations had the highest number of criteria met (averaged) of the 10 state regulations examined. We then describe states' regulations overall alignment to the Care Model; i.e., ranked from highest to lowest number of total criteria met across all nine recommendations of the Care Model. This is followed by descriptions of individual states' alignment to each of the Care Models' recommendations (Figure 2). Trends in alignment to specific recommendations and individual criteria met are described last.

### 3.1 Regulation alignment with the Alzheimer's association comprehensive care model

The research team analyzed regulations from California, Missouri, Minnesota, Indiana, Kentucky, New Mexico, Virginia, Texas, Georgia, and Connecticut. The search and analysis of state regulations collected 15 individual Titles across 10 states (Table 1).

Across the 10 examined states, ALF regulations aligned most to four of the nine Care Model recommendations: Supportive and Therapeutic Environment (4.2 average, out of 5 criteria), Information Education and Support (3.75 average, out of 5 criteria), Ongoing Care (7 average, out of 10 criteria), and Staffing (4.02 average out of 6 criteria). In contrast, states' regulations aligned less closely on average with Person-Centered Assessment and Care Planning (3.1 average, out of 5 criteria), Person-Centered Care (3.3 average out of 6 criteria), Transitions and Coordination of Care (2.8 average out of 5 criteria), Detection and Diagnosis (3.57 average out of 7 criteria), and Medical Management (3.5 average out of 7 criteria). The Information, Education, and Support recommendation showed the least variation where each state regulation met from 60% to 80% of criteria. Transitions & Coordination of Care and Supportive and Therapeutic Environment showed the greatest variation, with a range of 20% to 80% and 40% to 100% criteria met by each state's regulations.

#### 3.1.1 Recommendation: supportive and therapeutic environment

The Supportive and Therapeutic Environment Recommendation included five criteria with a high average alignment score of 84%, 4.2 of 5 criteria. Indiana, New Mexico, Missouri, California, and Minnesota regulations aligned with 5 of 5 criteria. Texas met 4 criteria, Georgia 3, and Connecticut 2. All 10 states met the “support courtesy, concern, and safety within the care community” criteria, while 3 more of the criteria were met by 9 of 10 states. The remaining and least met criterion was met by 6 states: “Create a sense of community within the care environment.”

Similarly, all states' regulations meet the Supportive and Therapeutic Environment criterion of encouraging care partners to work together and plan together. In line with this criterion, all states' regulations outlined the importance of communication between all ALF staff, guardians/family, and residents' clinicians when creating individual care plans or notifying relevant parties of major health events. Also in line with this criterion, all states required daily communication expectations or had communication protocol for all care staff working in ALFs. However, while communication, timely communication, and care partners were mentioned by name, these terms were often underdefined or went unclarified in regulations.

#### 3.1.2 Recommendation: information, education, and support

For this recommendation, all states' regulations meet the criterion to provide education and support early in the disease to prepare for the future. Specifically, states' regulations required direct care workers to receive education on how to best support the independence and care of residents early in their AD/ADRD diagnosis; and be trained to recognize and effectively manage early symptoms. This was with the caveat that training expectations be appropriate for the level of direct care workers' role or license/certification. Conversely, no state regulations addressed the criterion to use technology to reach more families in need of education, information, and support.

While states robustly aligned with the Information, Education, and Support Recommendation overall (74% average alignment), regulations among eight states still failed to comprehensively outline training content. Rather, the phrasing indicated that all such topics listed in the five criteria of this recommendation should be outlined in the training curricula. Regulations also outlined that specifically trained team members or licensed instructors teach the in-depth education indicated by the curriculum.

#### 3.1.3 Recommendations: ongoing care, staffing

The average alignment score for Ongoing Care and Staffing Recommendations was 70% across the states examined. Seven states meet over half of the 10 criteria here. Six of seven meet eight or more. A criterion with particularly low alignment from all states was that which would require regulation to recognize the significant investment required to implement non-pharmacological practices across different settings. Only California's regulations had provisions that address this criterion.

Of the Staffing recommendation, all states meet at least half of the six criteria. Criteria with which the fewest states' regulations met (only four states each) were the development of systems for collecting and disseminating person-centered information and promotion, and encouragement of resident, staff, and family relationships.

#### 3.1.4 Recommendations: person-centered care, detection and diagnosis, medical management

Care Model recommendations with an average of 55% of criteria met or less include Person-Centered Care (55%), Detection and Diagnosis (51%), and Medical Management (50%) (Figure 1). With respect to the Person-Centered Care recommendation, all states' regulation addressed its criterion of regularly evaluating care practices and making appropriate changes. No states' regulations contain additional language that would meet the criterion for staff to “build and nurture authentic and caring relationships.” Three states met the criterion to recognize and accept a resident's reality. At least 6 out of 10 states met the remaining criteria within the Person-Centered Care recommendation: know the person living with dementia, identify and support ongoing opportunities for meaningful engagement, and create and maintain a supportive community for individuals.

State regulations least met criteria for the Detection and Diagnosis, and Medical Management recommendations. All states' regulations did met two of the seven total criteria for Detection and Diagnosis: the first, listen for concerns about cognition, observe for signs and symptoms of cognitive impairment, and note changes in cognition that occur abruptly or slowly over time, and the second, develop and maintain routine procedures for detection of cognition and referral for diagnostic evaluation. While all state regulations met these listed criteria, Texas and Virginia meet less than half of the total for Detection and Diagnosis. Of the Medical Management criteria, four of ten states met less than half. No state regulations met two criteria: make information about brain health and cognitive aging readily available to older adults and their families, and encourage persons living with dementia and their families to start end-of-life care discussions early.

#### 3.1.5 Recommendation: person-centered assessment and care planning

All states but Georgia met at least half of the criteria in this recommendation, and all states' regulations contain language that addresses its criteria to perform regular, comprehensive assessments and timely interim assessments. All states' regulations also require the assessment of resident care plans at least annually, as well as requiring immediate assessment of the health and care plan upon a notable change in the character or health of a resident. Additionally, 9 of 10 state regulations meet the criterion to use documentation and communication systems to facilitate the delivery of person-centered information between care providers. Kentucky regulation did not meet this criterion. Despite the high number of states that met these two necessitate comprehensive care elements, no states met the additional criterion for the use of assessments as an opportunity for information gathering, relationship-building, education, and support of residents.

### 3.2 Care model alignment score differences by state

#### 3.2.1 California, Missouri, Minnesota: high alignment states

California regulation was the most aligned with the Care Model, with 44 of 56 criteria met (79%) (Figure 2). Excepting Staffing, California regulation met above the average number of criteria met for each of the nine recommendations. California's Staffing alignment score (4 out of 6 criteria met) was the same as the across-state average. Missouri and Minnesota regulations, second and third highest alignment states, followed a similar pattern of rank ordered recommendations the same as the across-state average rank order. Missouri and Minnesota had overall alignment scores of 75% and 71%, respectively.

#### 3.2.2 Indiana, Kentucky, New Mexico, Virginia: moderate alignment states

Ranked fourth and with an overall alignment score of 68%, Indiana regulation followed a similar form in the distribution of criteria met by top alignment states (Figure 2). Like California, Indiana's regulation met all criteria within the Ongoing Care and Supportive and Therapeutic Environments recommendations, and had 80% alignment to the Information, Education, and Support recommendation. Indiana also tied with California with 67% of Staffing criteria met. Indiana outperformed California in one recommendation, Person Assessment and Care Planning by here, Indiana meets. However, Indiana fell short of average criteria met within the Person-Centered Care (50%), Detection and Diagnosis (43%), and Medical Management (43%) recommendations. Kentucky (64%), and New Mexico (61%) the other states categorized as moderate alignment states, followed this same criteria alignment pattern across each recommendation.

#### 3.2.3 Texas, Georgia, Connecticut: minimum alignment states

Low alignment states, Virginia, Texas, Georgia, and Connecticut, range from a 52% to 59% average alignment to the Care Model (Figure 2). Connecticut has the lowest alignment at 52%. Connecticut regulation alignment to the recommendations of the Care Model mirrored states of moderate and high alignment. For example, the research team also observed a pattern of low average alignment in moderate and high alignment states in the Person-Centered Assessment and Care Planning and Detection and Diagnosis recommendation (68% and 57%). Connecticut had 80% and 71% alignment to the criteria in these recommendations, respectively. However, for Person-Centered Care and Ongoing Care, Connecticut's regulation has exceptionally low alignment at 30% and 33%, respectively. Moderate to high alignment states align more closely with the criteria in these recommendations, all meeting between 60% and 100% (Figure 2). Texas and Georgia did not meet a notably high number of criteria in, and specific recommendations of, the Care Model.

## 4 Discussion

Findings in this study provide new insight on the presence and absence of comprehensive memory care language in ALF regulation across states. The following provides an exploration of implications of these results and compares results with previous dementia and long-term care research. Implications of findings related to the Comprehensive Care model are first summarized, leading into a broader discussion of the role that comprehensive memory takes in ALF regulation.

### 4.1 Implications of low alignment to the comprehensive care model

States that failed to meet Information, Education, and Support criteria lacked training curriculum content or failed to define the unique supports needed in memory care. However, states that failed to specify training requirements in this area may rely on other resources. For example, a broad spectrum of NGO-driven caregiving guidance and model resources exist and which an individual facility may choose to implement. For example, the Alzheimer's Association has a Recognized Dementia Training Programs list that is easily accessible online (Alzheimer's Association, 2025). This study did not investigate evidence on the use or effectiveness or integration of these resources into state regulation. Many individual ALF providers may also have evidence-based care practices outlined within specific rules and regulations of individual facility sites and service organizations. This study did not measure comprehensive care practices of individual assisted living service organizations or facilities.

States' regulations did not consistently meet Transitions & Coordination of Care criteria. Outside of the Comprehensive Care Model used for this study, the Alzheimer's Association and other research suggest that care providers should be rehearsed in smooth care transitions for persons with cognitive impairment in order to avoid undue stress of residence (Hirschman and Hodgson, 2018). This evidence may support implementation of transitions and care coordination language in regulation as well. Though, additional evidence is needed to link regulatory standards to actual measured change in state-level care coordination practice in the ALF setting.

However, in the context of coordination of end of life care, (Temkin-Greener et al. 2023) findings revealed that assisted living communities are increasingly becoming facilities where residents remain until death. This evidence points to a need for increased direct care staff training overall for this type of care transition. Transitions such as hospice care without need for travel or large disruptions in routine are also preferred (Temkin-Greener et al., 2023). Given the choice, older adults, regardless of type of residence, prefer to receive hospice care where they currently reside (Pinto et al., 2024). This aligns with both the growing advocacy for aging in place (Owusu et al., 2023), and argument that assisted living facilities should emphasize residents' autonomy This is inclusive of residents' decisions to stay in place while transitioning into hospice care, wherever and whenever possible (Zimmerman, 2022).

Target areas for regulation change may also exist outside of mechanisms that govern the staff and services directly provided by ALFs. For example, specificity on the role of third party end-of life care exists within some states' ALF regulation. Regulation in this context may be essential in bridging the gap between residents' desire to die in place and direct care service limitations of their chosen ALFs. An examination of this regulatory approach includes a 2022 study of the presence of end of life care service regulation embedded in broader assisted living regulations of states. (Belanger et al. 2022) developed a cohort study of deceased assisted living residents in states with and without ALF regulation that explicitly mentioned private care aide, home health, or hospice care services. Findings revealed that states with more supportive text around these third party end-of life services were 1.46 times more likely to have had ALF decedents die in place between 2017 and 2019 (Belanger et al., 2022). This also aligns with integration of comprehensive language around transitions and care coordination in ALF regulation that the Care Model would require.

Overall, inconsistencies in state alignment to the Care Model also underscore the absence of a standardized enforcement mechanism across assisted living systems. Unlike federally regulated entities such as nursing homes, ALFs operate under decentralized state oversight. There is clear variation in training content and staff competencies. At baseline, all states allow full scope of care tasks of Certified Nursing Assistants (CNs) as is defined by 42 CFR § 483 (CFR Part 483, 2026) in the ALF setting. However, trained care providers still exist outside this guidance. For example, non-licensed certified providers such as care aides and activities directors. Previous study of care workers in ALFs that compared CN scopes of practice also confirmed the integral role care aides take on (McMullen et al., 2015). All state regulations analyzed permitted hiring of non-certified staff for direct care. For some states, particularly those with low overall Care Model alignment, the training topics required for these non-certified aids were minimal.

Regulatory enforcement mechanisms also differ substantially between federal and state oversight systems. Many states exhibit variability in enforcement protocols, including inspection frequency, penalties for non-compliance and compliance monitoring processes. In contrast, nursing homes subject to centralized federal oversights provide structured, standardized, and routinely monitored enforcement frameworks. What remains unclear is whether or how the variability of assisted living regulations found in this analysis are evidence that ALFs need homogenized oversight like that of nursing homes. Centralized oversight and standardized enforcement mechanisms may also not be an appropriate solution across diverse states.

As previously mentioned, Centers for Medicare and Medicaid Services (CMS) regulate nursing homes, requiring facilities to collect various data on the health and quality of life of residents. In contrast, and in the absence of uniform federal requirements outside of CMS-sponsored clinical setting encounters of residents, state agencies gather health and quality of life measures of ALF residents at individual discretion. Assemblage of such information may not consistently occur, can vary in methods of collection, and lack dissemination. This proves a challenge when attempting to compare existing data comprehensively and accurately across states, limiting our understanding of effective regulation. National studies driven by CMS, such as the National Health and Aging Trends Study or systematic gathering of Medicare and Medicaid Data often do not provide the granular detail needed to inform ALF-specific policy. The National Survey of Residential Care conducted by the CDC provided detailed insight yet was once and only conducted in 2010.

What can and has been systematically measured across states are the administrative data of Medicare beneficiaries living in ALFs (Thomas et al., 2018). Using Thomas's method, health outcomes and quality measures derived from ALF-residing Medicare cohorts can be linked to regulation. Existing examples include research publications referenced in Sections 4.1 and 4.2. Future study of regulatory presence of comprehensive practice in ALFs should be integrated with memory care quality measures drawn from Medicare administrative data. Pivotal work in this area include that of the multi-institutional Shaping Long-Term Care in America Project and its LTCFocus platform, who are collecting and disseminating market, health, and functional data related to long-term care practices and policies (LTCFocus, 2026).

### 4.2 The argument for universally required memory care training

The significance of state regulation requiring all direct care workers to participate in memory care training may be contextualized when paired with existing estimates that underdiagnosis is high in residential care (Tewari et al., 2025). Additionally, persons with an AD/ADRD diagnosis are at higher risk of experiencing certain adverse events. For example, risk of fall for those with diagnosis is over twice that of cognitively normal older adults (Kehrer-Dunlap et al., 2024). Connecticut, California, and Minnesota ALF regulations specifically require training in memory care (and the recognition of symptoms) for all direct care staff, regardless of whether an ALF's resident population or sub-population required memory care. However, individual ALFs may choose to exceed state requirements. This study did not explore factors of how, when, or which individual ALFs within the selected states do so.

The lack of dementia training for all direct care workers, not just those working in memory care units, in the remaining seven states of this study raises concerns about the quality and consistency of dementia-aware care provided to residents with mild or unnoticed cognitive impairment. There is a dearth of data that measures how well direct care workers can recognize and take appropriate action when community care-based residents show cognitive impairment symptoms.

It is also important to recognize that at a federal level, cognitive impairment and dementia care is part of certification curriculum Certified Nursing Aide (CNs or CNAs). Outside of re-certifications required every 2 years, retention of cognitive impairment knowledge and care practices may be dependent on the regulations and regularity specific to direct care staff training in ALFs and organization-level training requirements.

This highlights the need for an integrated workforce and quality oversight mechanisms. Current state regulations often separate staffing minimums from training content, creating fragmented accountability systems that fail to capture the cumulative impact on resident wellbeing. Regulation that jointly addresses staff sufficiency, competence, and care outcomes could better reflect the holistic demands of dementia care.

Training for all ALF direct care workers could prompt better recognition and timely adjustments in care for all ALF residents regardless of memory care placement. It is well-documented that older adults, in general, regardless of setting, are at an increased risk of developing an AD or ADRD. Because ALFs are home to a population with additional increased risk of developing cognitive impairment and can house residents in initial stages of cognitive impairment, direct care staff of ALFs should know how to recognize and manage symptoms across a spectrum from mild cognitive impairment to late-stage dementia. This sentiment is broadly shared by the NIH, the World Congress of Gerontology and Geriatrics, and the Alzheimer's Association (Alzheimer's Association, 2020; Morley et al., 2015).

Additionally, with the continued shortage of older adult care workers, it is prudent that the staff of an ALF be capable of switching between memory and non-memory floors and units. Universal memory care training would provide staff with a greater range of abilities to manage diverse care situations and residents' needs. This is not an endorsement to overwork existing care providers, but rather an emphasis on the importance of requiring evidence-based and universal dementia training to enhance workforce efficiency, adaptability, and care quality. Cross-training all direct care staff workers in memory care principles would better allow facilities to deploy efficiently across units, reducing disruptions in care and minimizing reliance on agency or temporary workers (Gkioka et al., 2020). Future research could explore whether states with universal dementia training requirements demonstrate a lower turnover rate or higher staff confidence in managing behavioral symptoms, which are two areas that significantly impact care quality, and facility stability (Aiken et al., 2023).

Evidence for targeting change in assisted living regulatory mechanisms specifically related to staff includes the 2021 research of Thomas et al. Here, changes in regulations governing direct care staff in ALFs from 2007 to 2018 were systematically compared to monthly hospitalization rates among assisted living residents. Among this sample was also a subgroup of residents living with dementia. An increase in specificity of the role and ratio of unlicensed direct care workers was associated with a 4% reduction in risk of resident hospitalization. An additional 2% point reduction was observed among the dementia subgroup (Thomas et al., 2021). This supports the argument for greater universal dementia care training among unlicensed workers. Findings also support the argument that that aides are essential drivers of quality in a diverse array of essential daily supports for residents. Hospitalization risk was oppositely correlated with increased regulatory specificity of licensed care workers (Thomas et al., 2021).

### 4.3 Limitations

The research team limited analysis to 10 states but data from this study indicates additional research of all 50 states is needed. Future findings using this Care Model will and should be compared against other evidence-based dementia care models. Building research from the model used in this study and others will both increase validity of findings and expand possible methods of analysis.

Additionally, ALF licensing structure within Connecticut regulation is unique among states. Other states include rules on built environments within the same regulations on ALFs and memory care in ALFs. In Connecticut, licenses are granted only service providers by the Assisted Living Services Agency. This is similar to the third party end of life care regulation mentioned in Section 4.1. In Connecticut regulation, housing facilities are considered separate entities from the service provider. Meaning, physical facilities are not under service provider jurisdictions. As a result, analysis did not include Connecticut's rules that addressed the built environments where assisted living services are provided. This likely contributed to the state's low average alignment to the Care Model, and very few criteria met within the Supportive and Therapeutic Environment recommendation (two of five).

Adverse events and care quality indicators were not included in analysis, though regulation can be directly linked using different and additional methodology. However, existing measures in broader LTSS research are not comprehensive, nor are they completely accurate representations of care quality. For example, nursing homes and home care based models are subject to robust quality measures developed over multiple iterations and implementations of evidence-based regulation dictated by CMS. To measure the effectiveness and quality of ALF regulation more accurately, researchers should use a greater diversity of care measures in order to account for differing regulatory standards across states. This might include both a systematic comparison of currently existing ALF data at the state level such a what can be from Medicare records, or novel projects that collect new, standardized data across states. Example measures might include facility payroll reporting to reveal resident monitoring behavior of workers, or a consistent across-state list of self-reported measures, such as the rate of pressure ulcers in residents.

Existing efforts here include both the Long Term Care Data Cooperative and LTCFocus research groups at Brown University.

### 4.4 Closing commentary

Solidifying comprehensive care practices in regulation comes with both potential benefits and challenges. Adding specificity to regulation can influence real-world changes in the quality of ALF services in memory care and improve outcomes among people with dementia in these settings (Ramesh et al., 2024). However, increasing training requirements also has the potential to further burden the care workforce and exacerbate workforce shortages, as well as create additional administrative and cost burden related to the enforcement of such requirements.

Regardless, this study provides a snapshot of the 2020 memory care environment in assisted living facilities across 10 states. In part, this project reflects the lack of existence and accessibility to consistent and facility-reported data on care quality in ALFs. In the context of the lack of consistently gathered data, regulation documents were a source for investigation that was both accessible and definitive. This provided the timeliness, accessibility, and detail required to evaluate memory care in ALFs in a comprehensive and novel way. Evaluation of regulation also allow for statistical and other methods of data analysis to function as supplementary evidence in future research. The methods of this study are building blocks from which researchers may build additional mixed methods to systematically collect and analyze data for future across-state LTSS studies. Ongoing effort here includes further definition and evidence for accurate, comprehensive memory care measures (Zimmerman and Fazio, 2022), and increased support to establish dementia care data sharing networks and standards across states and institutions (Research Network for Alzheimer's Disease Home and Community Based Services, NIH RePORTER, 2025).

## Data Availability

The raw data supporting the conclusions of this article will be made available by the authors, without undue reservation.
